# Inequalities in COVID-19 impact on preschool mental health in India: key moderators of adverse outcome

**DOI:** 10.1136/bmjph-2024-001209

**Published:** 2024-12-12

**Authors:** Helen Sharp, Nicky Wright, Laura Bozicevic, Thirumalai Ananthanpillai Supraja, Andrew Pickles, Jonathan Hill, Prabha S. Chandra

**Affiliations:** 1 Department of Primary Care and Mental Health, University of Liverpool, Liverpool, UK; 2 Department of Psychology, Manchester Metropolitan University, Manchester, UK; 3 Department of Psychiatry, Manipal Academy of Higher Education, Manipal, India; 4 King's College London Institute of Psychiatry Psychology & Neuroscience, London, UK; 5 National Institute of Mental Health and Neurosciences, Bangalore, India

**Keywords:** COVID-19, Mental Health, Public Health

## Abstract

**ABSTRACT:**

**Introduction:**

Worldwide research suggests that the COVID-19 pandemic had little to no overall effect on preschool children’s mental health, but that the impact is variable depending on pre-existing and COVID-19-related inequalities. Evidence from low- and middle-income country settings is sparse, yet effects may be more variable due to greater inequalities. We provide the first empirical evidence for the impact of the pandemic on emotional and behavioural problems in Indian preschool children, after accounting for normative age-related change, and test whether the impact varied depending on COVID-19-related inequalities.

**Methods:**

Families participating in an Indian-based prospective longitudinal birth cohort (Bangalore Child Health and Development Study) provided data at age 2 years (before COVID-19) and again during COVID-19 (n=528). Mothers reported child emotional and behavioural problems and a range of COVID-19-related adverse experiences.

**Results:**

There was a small overall pandemic effect on emotional (rate ratio (RR)=1.31, p=0.040), but not behavioural problems, after adjusting for age-related change. However, compared with the lowest risk level, emotional and behavioural problems rose higher compared with whole sample age-expected rates in families who reported the highest levels of perceived negative impact of COVID-19-related adversities (moderation p<0.001, RR=2.43 and p<0.001, RR=1.32), COVID-19 life events (p<0.001, RR=3.28, and p<0.001, RR=1.26) and time the child spent playing alone (p<0.001, RR=2.49). Emotional problems rose higher with high perceived COVID-19 maternal stress (p=0.013, RR=1.57) and with increased child mobile phone use (p<0.001, RR 1.48). Secondary analyses controlling for variation in age trends within moderator subgroups revealed these to be rarely significant. Where significant and accounted for, having more children living at home emerged as protective, whereas living below the poverty line emerged as a risk for adverse pandemic impact on child mental health.

**Conclusion:**

A small overall increase in preschool mental health problems was evident. However, this masked substantial worsening of such problems in families with elevated COVID-19 adversities in India. These findings can inform the targeting of policy and practice initiatives to better mitigate adverse longer-term mental health outcomes arising from the pandemic response.

**WHAT IS ALREADY KNOWN ON THIS TOPIC**

- Worldwide research suggests that the COVID-19 pandemic had little to no overall effect on preschool children’s mental health, but that the impact is variable depending on pre-existing and COVID-19-related inequalities. However, very few studies have compared levels of emotional and behavioural problems prior to and after the onset of the pandemic, and no studies have accounted for normative age-related changes in symptoms over time. Evidence from low-middle-income settings is sparse, despite the greater impact that COVID-19 had in these settings on adults, and on family circumstances which are widely reported, but not researched systematically.

**WHAT THIS STUDY ADDS**

- This study reports evidence that, after accounting for normative age-related change in emotional and behavioural problems, the impact of the pandemic on Indian preschool children was markedly worse for children living in families who faced higher perceived negative impact of COVID-19-related adversities and COVID-19-related life events, where mothers perceived high stress due to the pandemic, and for children who spent more time playing alone and using mobile phones. Accounting for significant variation in age trends for moderator subgroups indicated that the effects of the pandemic were also worse for those living below the poverty line and that having more children living at home was protective, possibly mitigating against child social isolation.

**HOW THIS STUDY MIGHT AFFECT RESEARCH, PRACTICE OR POLICY**

- Policy initiatives and school mental health programmes should be designed to identify ongoing emotional and behavioural problems postpandemic and to reduce them to prevent exacerbating India’s mental health burden for years to come.

## Introduction

A small number of studies worldwide have provided evidence regarding the impact of COVID-19 on preschool-aged children’s mental health by comparing levels of emotional and behavioural problems prior to and after the onset of the pandemic.^1^,^4^ Collectively the evidence suggests little to no overall effect of the pandemic on young children’s mental health, but that the impact is variable depending on pre-existing and COVID-19-related inequalities. However, the interpretation of whether there has been a COVID-19-related change needs to take into account of naturally occurring developmental progressions of child symptoms over early childhood. No previous study has done this. There is also very little empirical evidence from low- and middle-income countries (LMICs) on the impact of the pandemic on children’s mental health,^5^ despite the greater impact of COVID-19 in these settings on adults and on family circumstances.^6^,^8^ The impact of the sudden lockdown in the first wave of the COVID-19 pandemic on income and housing in many Indian families has been well documented, with low-income urban families experiencing a disproportionate impact.^9^

In the following study, we report findings from families living in urban Bangalore. To set the context, on 25 March 2020, the government of India imposed a sudden complete nationwide lockdown for 21 days, with the closure of non-essential markets and a complete halt to all national rail networks and international and domestic flights. These restrictions were particularly challenging for daily wage workers and migrant labourers, especially in large cities like Bangalore, as many such families were reliant on these markets and local travel for their livelihoods. With COVID-19 restrictions, many could not afford to stay in the city and moved back to their native villages leading to displacement and families being split, sometimes with half the family staying and the other half moving to native villages. Alongside the health effects during the first wave, economic effects were enormous. Families living in slums in urban Bangalore faced serious hardship. Their incomes fell as most of them were daily wage earners. One study from Bangalore found that slum residents spent their savings, reported food insecurity and borrowed money.^10^ According to this study in the first month after the lockdown began, roughly 50% of household heads in Bengaluru had lost their primary source of income. After the lockdowns ended, people faced widespread job losses and wage reductions. By mid-November 2020, one-quarter of prepandemic income in Bangalore had still not been recovered. In the first wave in addition to lost livelihoods, stigma and fear played a major role in increasing distress. In a study conducted in South Bangalore (where the majority of families from our cohort live) more than half of the participants reported that some people have refused to visit their home even after the person with COVID-19 recovered, more than one-quarter of the participants reported that they would possibly prefer others not knowing about their infection to others.^11^ More than half of the participants reported that they felt ashamed or embarrassed due to their COVID-19 infection. By the time the second wave came, stigma had come down but poverty and food insecurity continued. Bangalore saw a large number of deaths and hospitalisations, with a lack of beds being a major source of stress and fear. Social distancing was practised widely in Bangalore because of the overall better educational status of the residents and the educational information provided by the government. However, this was challenging in urban slums where there continued to be overcrowding and difficult living arrangements. Finally, for children, government-funded Anganwadi centres support families of preschool children including providing nursery care closed. While many Indian schools (including primary schools) moved to online classes in the later part of 2020, children from low-income families were left behind because they did not have access to the internet or smartphones and laptops. Most children in our cohort were due to start primary school in 2020 but were not able to do so because of admission closures and loss of livelihoods which led to parents not being able to afford education.

In this study, we examine the impact of the COVID-19 pandemic and potential moderators of impact on preschool-age children’s mental health after accounting for naturally occurring developmental changes in an epidemiological cohort of children from urban Bangalore in South India. Families reported on child emotional and behavioural problems before and during COVID-19. We examined for moderation of impact by COVID-19-related life events, perceived impact of COVID-19 across multiple domains of day-to-day life and perceived stress. We also examined moderation by factors identified in empirical and opinion literature on the impact of the COVID-19 pandemic in India; job loss, home overcrowding, prior domestic violence, deprivation, gender, increased child mobile phone or TV usage and increased time spent alone.^12^,^16^ We hypothesised that the impact of the pandemic would be variable depending on inequalities, with children in families more adversely affected by the pandemic showing the greatest increase in emotional and behavioural problems after accounting for normative developmental changes.

## Methods

### Study design and sample

The Bangalore Child Health and Development Study (BCHADS) is a prospective longitudinal birth cohort established to examine the prenatal and infancy risk and protective factors for child mental health.^17^ 909 families were recruited in pregnancy (at either the first or the second trimester of pregnancy) from low-income areas of urban Bangalore. All participants were recruited when attending antenatal appointments in a Government Referral Hospital—Urban Primary Health Centres (UPHCs) at Banashankari, N.R. Colony, and at Siddaiah Road between July 2014 and November 2016. The sites were chosen as they provide free healthcare to pregnant women and cater to women from different resource settings as we aimed to represent the general population and are also reachable within an hour from the study site (National Institute for Mental Health and Neuroscience). All women attending antenatal appointments in the three maternity services were approached consecutively and were initially screened by the team of research staff. Those women who met the inclusion criteria (ie, not reporting major mental illness such as psychotic disorder, or major health complications during the current pregnancy, or harmful use of alcohol or other psychoactive substances, who spoke the language for assessment, and who planned to reside in Bangalore) were invited to participate in the study. Overall, 1048 women were screened. Only one was excluded on health grounds because we set the threshold high in order to retain as many as possible of those with health risks of relevance to the study. A further 85 were excluded because they did not speak Kannada, 30 planned to move elsewhere, and 23 did not consent, resulting in a sample size pregnancy of 909.

Of these 909, 84 women were excluded for the following reasons: 44 because the pregnancy had a poor outcome (ie, miscarriage, medical termination, intrauterine death) or became obstetrically high risk later in pregnancy (eg, gestational diabetes), 5 because of stillbirth, 24 because of neonatal death (under 1 month), 6 because of infant death (under 1 year), and 5 because infants were twins. Mothers completed assessments at 8 weeks, 6 months, 1 year, and 2 years old (last wave January 2017–December 2019). A COVID-19 follow-up was conducted from July to September 2020 (age 2.5–4.5 years; mid-pandemic), and at this stage, families were approached in random order. We considered the possibility that families facing the COVID-19 adversities to be studied might have taken longer to contact, in which case the child age at assessment could have been conflated with vulnerability associated with COVID-19 adversities. We therefore computed correlations between child age and the vulnerability variables (ie, COVID-19 impact scores, COVID-19 stress and COVID-19 stress event), and in each case, correlations were below 0.10 and entirely non-significant.

677 families gave data at age 2 (677/810; 83.6% of those eligible to be approached for the follow-up) and 585 (585/723; 80.9% of those eligible to be approached for the follow-up) gave data mid-pandemic (online supplemental figure S1 in online supplemental appendix 1). 585 provided CBCL data at either time point (585/825; 70.9% of those eligible at birth for follow-up) and form the sample analysed here. Of these, 532 gave prepandemic CBCL data, 583 gave CBCL data at mid-pandemic, 530 gave data at both timepoints, 2 had prepandemic only and 53 gave mid-pandemic CBCL data only.

At the age of 2 years, mothers and children completed lab and home assessments during which developmental assessments and maternal reports of child mental health were gathered. Telephonic interviews were completed during COVID-19.

### Measures

#### Sociodemographic variables

Maternal age, parity, education, religion and family income (categorised using the Kuppuswamy Index)^18^ were recorded at recruitment. During COVID-19, we recorded child age, total number of children in the household (continuous variable); ownership of Below Poverty Line (BPL) card indexing deprivation (0=no, 1=yes) and child gender (0=male, 1=female).

#### Child emotional and behavioural problems

The internalising and externalising problems syndrome scales from the preschool Child Behavior Checklist—CBCL 1½−5^19^ were used. The full CBCL was collected at age 2, but to reduce participant burden during the mid-pandemic assessment a subset of 15 externalising items and 13 internalising items were used (online supplemental appendix 2 describes item selection using confirmatory factor analysis on the age 2 BCHADS data and a UK sister cohort at age 5 years). Analysis used subscale scores from the shortened externalising and internalising scales which showed acceptable to good internal consistency at age 2 (α=0.82 and α=0.68) and good internal consistency at mid-pandemic (α=0.83 and α=0.80). The strength of association between the shortened CBCL subscales and the full subscales administered at age 2 was high for internalising (rho=0.84, p<0.0001) and externalising scales (rho=0.96, p<0.0001), supporting the use of the short form in analyses from both time points.

#### COVID-19-related adversities

Two measures used to assess pandemic-related difficulties described below were developed by our team on the basis of the literature available at the time, typically opinion pieces or letters to journals, on factors that might have influenced family life in India (COVID-19 Impact Scale, COVID-19 Perceived Stress Scale). In addition, we adapted measures developed or used in the BCHADS sister cohort in the UK, the Wirral Child Health and Development Study (The Lockdown Perception Scale, COVID-19 Life events, Changes due to COVID-19). Each scale is described briefly below. Scale items are given in online supplemental appendix 3, online supplemental table S1).

#### COVID-19 Impact Scale

We developed this scale to assess the impact of the pandemic on the availability of food, medicine and healthcare, and on family members’ relationships and stress levels (6 items rated; not at all (0), A little (1), Moderately(2), Severely (3)) and changes in the home environment (noise and overcrowding; plus 2 items rated Less than usual (0); The same(1); More than usual(2)). Internal consistency was good (α=0.70) so total score was used in analyses. Item total correlations ranged from 0.02 to 0.60, though since alpha would only be marginally improved to 0.75 with the removal of the two low items (items pertaining to overcrowding, noise levels) they were retained to ensure broad coverage of COVID-19 impact.

#### COVID-19 Perceived Stress Scale

We developed this scale to assess maternal perceived stress due to the pandemic using three items (rated not at all (0), A little (1), Moderately (2), Severely (3)). The internal consistency was good (α=0.82), and a total score was used in analyses. Item total correlations ranged from 0.62 to 0.74.

#### Lockdown perception

Lockdown impact was assessed using one question developed for the COVID-19 Household Survey by researchers at the University of Liverpool. Mothers indicated whether the effects of lockdown were entirely negative, both positive and negative, more positive than negative or hadn’t really changed their life. A binary variable reflecting 1=entirely negative, 0=other three responses was used for analysis.

#### COVID-19 life events

The presence or absence (0=no, 1=yes) of 20 stressful events related to COVID-19, adapted from Wright *et al*,^20^ were summed to create a total life events score.

#### Changes due to COVID-19

Questions were developed to assess job loss of both father and mother (binary variable 0=no loss or one parent, 1=both parents), and changes in children’s habits compared with before the pandemic in playing alone, watching TV and using a mobile phone. Rates (less than usual, same as usual or more than usual) were collapsed into binary variables for analysis (0=same/less, 1=more).

#### Previous partner violence

Domestic family violence from birth was reported by mothers at the 2-year assessment using the 18-item Indian Council of Medical Research Domestic Violence Assessment Questionnaire^21^ describing different forms of physical violence. A binary variable reflecting 0=no violence, 1=presence of violence was generated for analysis.

### Statistical analysis

To analyse the change in CBCL scores from the age 2 assessment to the follow-up during COVID-19, we used a random effects repeated measures model for responses with a skewed distribution (overdispersed Poisson). A simple comparison of emotional and behavioural problems for the cohort before and during the pandemic would conflate the considerable age-related changes expected from typical development with the changes specific to the onset of the pandemic. Our analysis decomposes the overall change with time into these two components. The basic model included a covariate for the age-at-assessment, a dummy variable for the pre–post pandemic comparison, a Gaussian random intercept to account for the overdispersion and correlation over time in symptom scores, and used a log-link function (the canonical link for a Poisson response) implying that all effect estimates were multiplicative (thus ensuring positive symptom predictions). The effect of moderation of the impact of the pandemic was explored by including the moderator as both a main effect on the responses and as an effect specific to the pandemic period. This took account of the possible differences in the reported symptoms before the pandemic of those children whose parents reported more or less of the potential moderator. For example, mothers reporting stress during the pandemic might also have reported more child problems prepandemic. All analyses were undertaken in Stata V.18^22^ using the gsem command^23^ with the systematic effects of covariates, dummy variables and moderators displayed graphically, and estimated pandemic effects with 95% CIs and Wald tests of moderation displayed using forest plots. Effects are reported as rate ratios (RRs). For clarity of exposition for non-binary moderators, we display the estimated effect for those in the lowest and highest categories. Online supplemental appendix 4 gives a more technical model description, and online supplemental appendix 5 details the results of a set of models that were re-estimated with the inclusion of moderator-by-age interaction terms as a possible confounder of moderated pandemic effects.

Models were estimated by maximum likelihood and thus included all participants with either a prepandemic or postpandemic CBCL score. We report findings from logistic regression of baseline factors associated with dropout from cohort inception to that included in this sample. Additional missing data for the moderator variables were minimal (less than 3%).

## Results

### Descriptive statistics

At recruitment, all mothers were married, around half were multiparous, just under half had a family income that placed them in the low socioeconomic status (SES) range for India, and only one-third of the sample was educated beyond secondary school (table 1). Online supplemental table S2 (online supplemental appendix 6) shows the comparison between demographic characteristics of women included in the analyses (n=585) with those excluded from the analyses (n=324). Our cohort population is comparable to the population of the same region (Karnataka) whose data are provided by the 2015–2016 National Family Health Survey (NFHS-4) (IIPS and ICF, 2017).^24^ Our cohort is similar in terms of education (72% of Karnataka women’s age 15–49 are literate—that is, have either completed at least standard six or passed a simple literacy test), religion (75.2% of the women in the urban areas are Hindu) and Body Mass Index (22.7% of currently married women in Karnataka are underweight). The proportion belonging to the BPL socioeconomic group was similar to the 13% seen in Karnataka urban areas. For the analysed sample, mean externalising and, to a lesser extent, internalising scores decreased from age 2 to 4.5 years (table 1). High rates of job loss were experienced by one parental figure during the pandemic (69.2% fathers and 49.9% of mothers). Job loss of both parents was experienced by 21.7% of the sample. Over a third rated their experience of the lockdown as ‘entirely negative’ (41.3%).

**Table 1:** Demographic characteristics of the sample at initial recruitment in pregnancy and descriptives of the variables assessed prepandemic and postpandemic

Demographics	N. total sample	% (N)	
SES at recruitment	533*		
(Low-SES/Below poverty)		8.4 (45)	
(Upper Low-SES)		45.2 (241)	
(Middle-SES)		43.2 (230)	
(High-SES)		3.2 (17)	
Education (above secondary)	584	31.8 (186)	
(up to secondary)		68.2 (398)	
Religion	581		
(Hindu)		84.2 (489)	
(Muslim)		15.5 (90)	
(Christian)		0.3 (2)	
Marital status (married)	578	100.0 (578)	
Parity (primiparous)	569	44.5 (253)	
Child sex (female)	585	51.1 (299)	
		M (SD)	Range
Maternal age	585	22.89 (3.835)	18–36
Child age pre-pandemic (months)	528	23.87 (1.90)	21–36
Child age post-pandemic (months)	528	53.00 (8.00)	39–68
Behavioural problems		M (SD)	Range
Externalising problems pre-pandemic	532	8.76 (6.00)	0–27
Internalising problems pre-pandemic	529	2.77 (2.82)	0–15
Externalising problems during-pandemic	583	5.37 (4.39)	0–21
Internalising problems during-pandemic	584	2.58 (3.11)	0–17
Moderators		M (SD)	Range
COVID impact	582	6.76 (3.22)	1–18
COVID stress	582	5.40 (2.75)	0–9
COVID events	583	3.91 (2.62)	0–15
N. children at home	582	2.20 (1.18)	0–10
		% (N)	
Perception of lockdown (entirely negative)	583	41.3 (241)	
Report of domestic violence in the first 2 years postpartum (yes)	585	14.5 (85)	
Job loss†			
(Yes - both parents)	571	21.7 (124)	
(Yes – mother)	337	49.9 (168)	
(Yes – father)	555	69.2 (384)	
BPL card‡ (yes) – during COVID	583	65.0 (379)	
Child watching TV (more)	574	55.1 (316)	
Child use of mobile phone (more)	582	63.2 (368)	
Child playing alone (more)	584	16.8 (98)	

* SES at recruitment was based on the Kuppuswamy Index. Missing data are due to some women not knowing the family income on which this information was based.

† Question concerning job loss only asked for participants who were in employment at the start of the pandemic.

‡ BPL card: Family have Government Below Poverty Line (BPL) card.

SESsocioeconomic status

### Change in child mental health problems from prepandemic to during the pandemic

**Figure 1:**
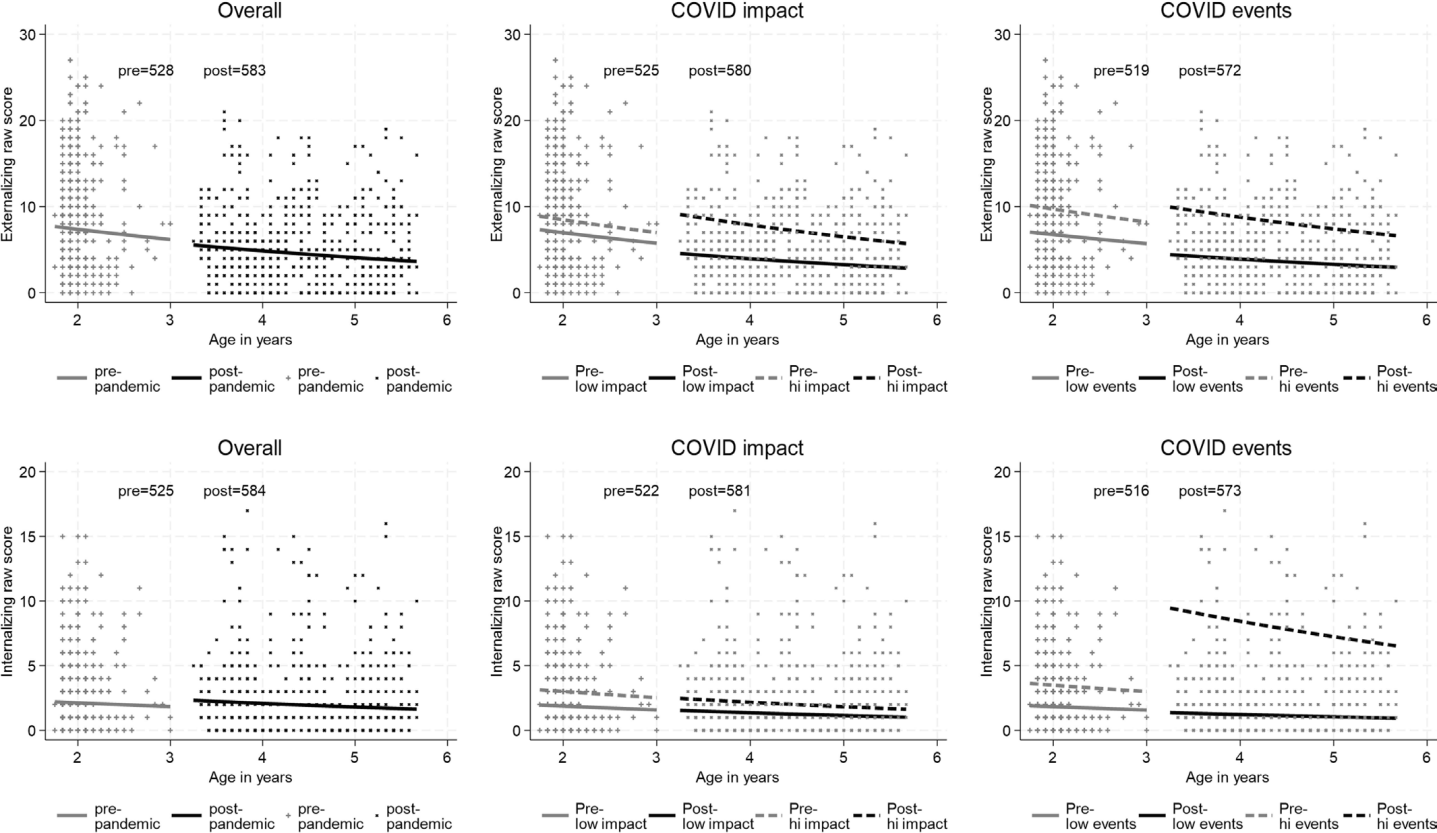
Model estimates of the decomposition of the change in child symptoms due to maturation and onset of the pandemic, overall and in COVID-19 impact groups. Note: The left-hand panels of the figure show the model estimated systematic decline in symptoms with age within the cohort prepandemic and during the pandemic, with a disjunction between the two reflecting the estimated impact of the pandemic.

The panels of figures1  2 show scatter plots of the distribution of internalising and externalising raw CBCL scores prepandemic and during the pandemic. These scores are all zero or greater and positively skewed. The effect of assessing at a defined point during lockdown led to a wide age range, evident in the wider spread of ages at assessment mid-pandemic. Figure 1 shows the model estimated systematic decline with age within the cohort prepandemic and during the pandemic, with a disjunction between the two reflecting the estimated impact of the pandemic. Adjusting for age, there was no effect of the pandemic on externalising (simple RR: 0.613, p<0.001; age-adjusted RR 0.936, p=0.452) and a small and marginally significant (taking into account multiple testing) effects on internalising (simple RR 0.920, p=0.026 and age-adjusted 1.307, p=0.040 respectively).

**Figure 2:**
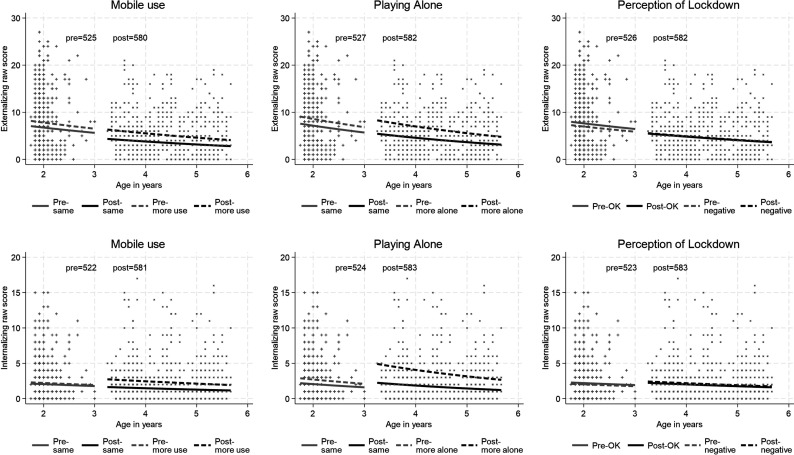
Model estimates of the decomposition of the change in child symptoms due to maturation and to the onset of the pandemic, in high and low mobile phone use, playing alone and negative perception of lockdown groups.

### Moderators of the change in child problems

**Figure 3:**
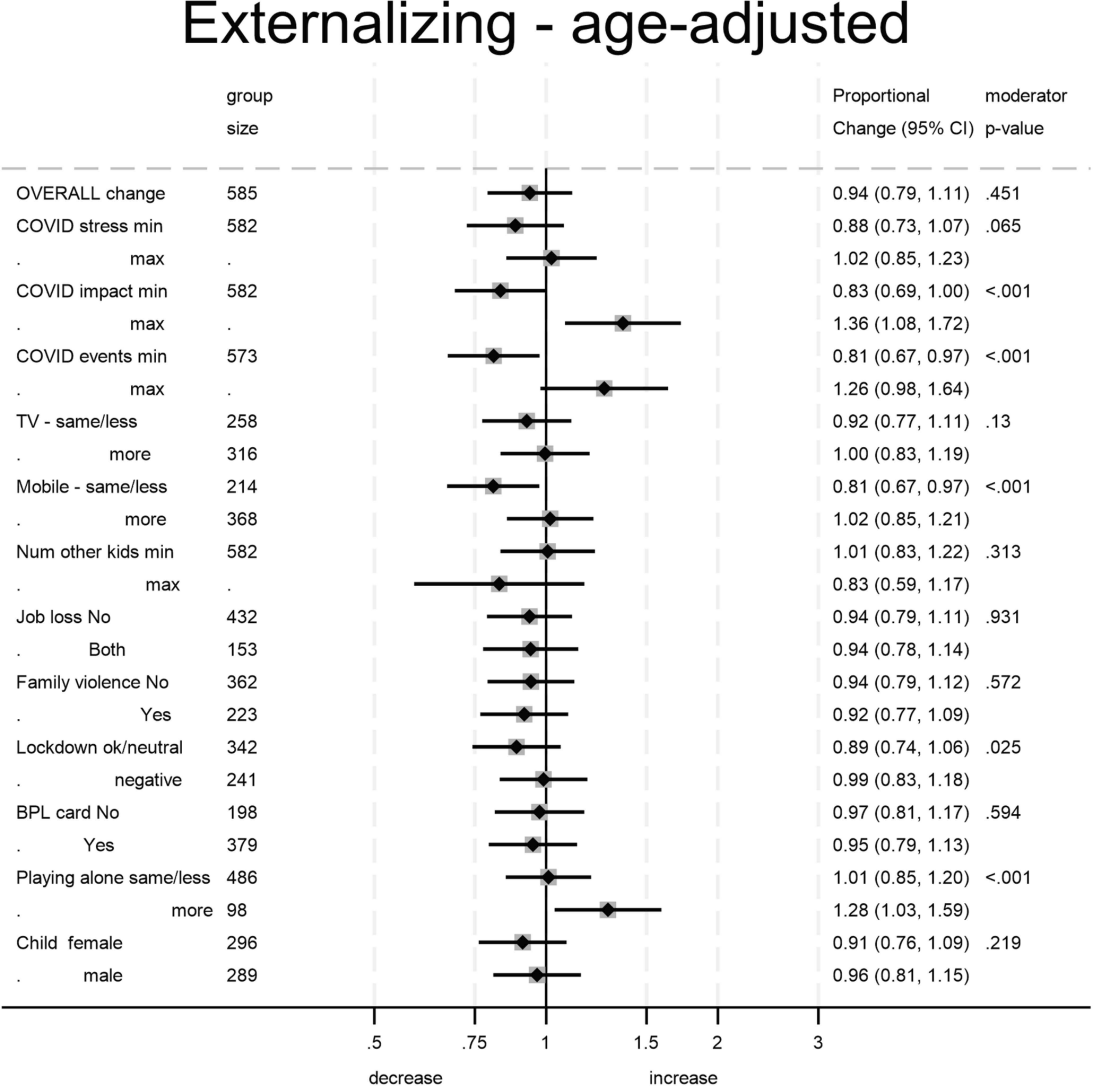
Estimates of the change in child externalising problems attributed to the pandemic. For moderators treated as continuous group size denotes overall sample size. The moderator p value denotes the significance of the moderator on the pandemic effect and not the significance of the difference from the no-pandemic effect (null value=1) for the subgroup.

The moderator analysis showed that the overall effects appear to hide some marked divergence between moderator groups. Our examination of 12 potential moderators found 4 significant (all 4 after Bonferroni correction) for externalising symptoms and 6 (5 after Bonferroni correction) for internalising symptoms. Figures3  4 show the pandemic-associated change estimates for the lowest and highest category of each moderator and the CI for each of these estimates.

**Figure 4:**
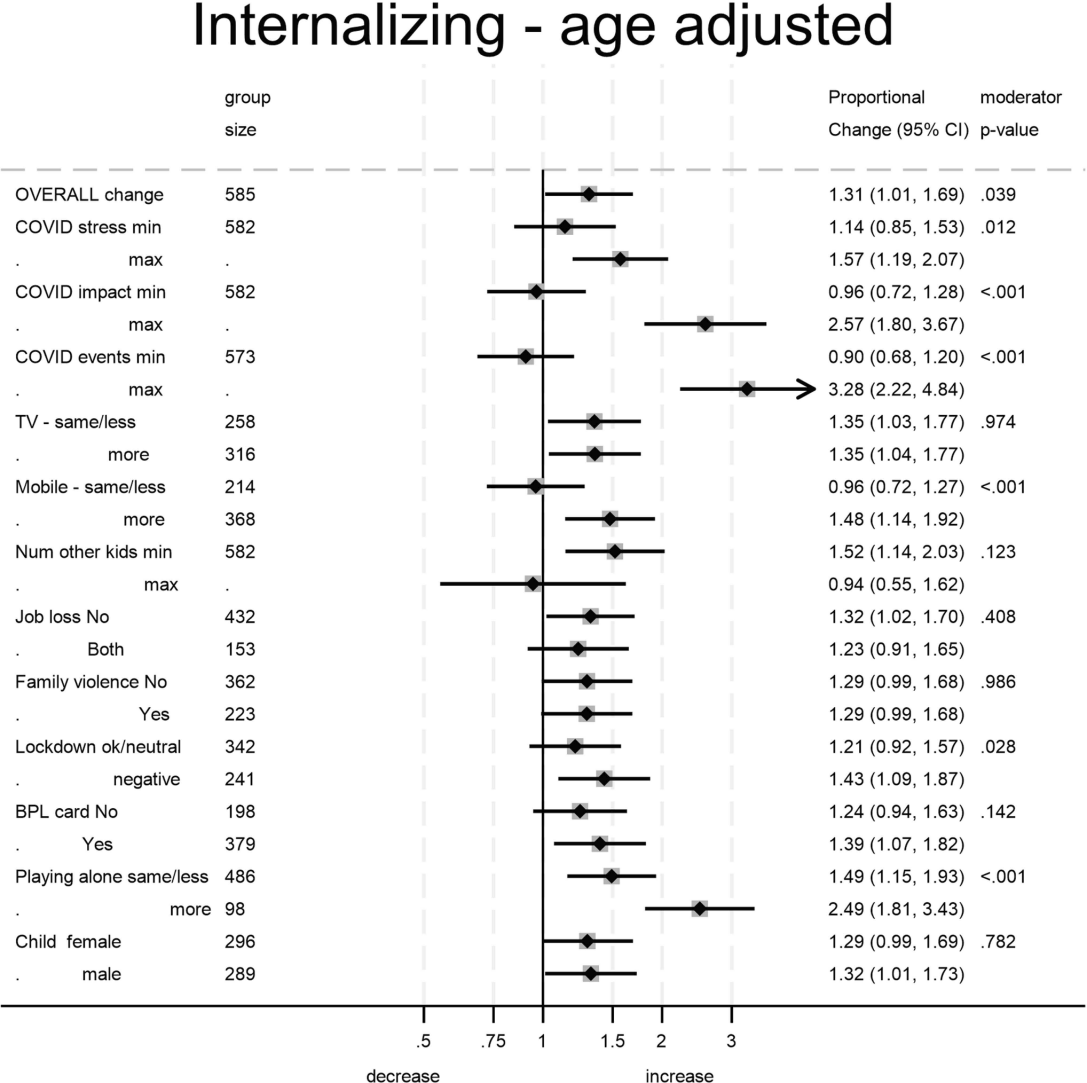
Estimates of the change in child internalising problems attributed to the pandemic. For moderators treated as continuous group size denotes overall sample size.The moderator p value denotes the significance of the moderator on the pandemic effect and not the significance of the difference from the no-pandemic effect (null value=1) for the subgroup.

In figure 1, it can be seen that mothers who reported a greater impact of the pandemic had reported higher levels of child mental health symptoms prior to the pandemic. Moderation by impact taking account of this difference is nonetheless evident in the widening of the gap between the age-related symptom levels. Where mothers reported high COVID-19 impact the child symptoms that they reported during the pandemic rose still higher (compared with whole sample age-expected rates) while those who reported no impact actually reported a reduction in child symptoms. IQR effect estimates, comparing those at the 75th percentile of the COVID-19 impact measure with those at the 25th percentile, showed a further 16% greater increase in externalising symptoms and 34% increase in internalising symptoms. The corresponding estimate for the COVID-19 stressful life events was 9% for externalising and 29% for internalising. Perceived maternal stress (online supplemental appendix 3, online supplemental figure S2) was associated with increases in reported internalising symptoms from similar prepandemic levels, with those at the 75th percentile showing a 20% increase.

In figure 2, the difference in child mental health symptoms reported for children who played with a mobile phone more often during the pandemic, compared with those not doing so, grew larger, and rates decreased among low users for externalising symptoms (21% decrease) but increased among higher users for internalising symptoms (52% increase). Increased time playing alone was associated with increased reported rates of both externalising (27% increase) and internalising symptoms (100% increase) with little change among those whose play was not so affected. The two plots for significant moderation by lockdown perception (which did not survive correction for multiple testing) show the differences both before and after to be slight. Pandemic-associated change in child symptoms was not related to increased TV watching, number of other children in the household job loss, past domestic violence, deprivation as indicated by BPL card nor the gender of the child.

### Secondary analyses: addressing possible confounding by subgroup-specific age trends

In response to a reviewer’s comments, we conducted analyses to test whether the background developmental age trends in scores might differ across the moderator subgroups. Such effects could be potent confounders for our moderated pandemic effect estimates. We therefore reran each model with an additional age by moderator interaction term to account for any potential differences in age effects within moderator subgroups. Scatter-line plots display the age trends and pandemic shift and forest plots of subgroup pandemic effect estimates and are shown in online supplemental appendix 5. Consistent with the age plots, significant age-moderation was limited to only 3 of the 12 moderator variables (number of other children living at home (p=0.036 for externalising symptoms, p=0.022 for internalising); increased TV watching (p=0.042) and BPL card (p=0.009) for internalising symptoms)).

After including the interactions to factor out any differential age-expected trends, the overall pattern/direction of pandemic effects remained the same. The larger SEs compared with the size of estimates means that for externalising behaviour, moderation by COVID-19 impact (p=0.006; increase over IQR effect=44%) stayed significant, while lower scores for those for maternal COVID-19-related perceived stress (p=0.018; increase over IQR effect=45%) and a higher number of other children living at home became significantly protective (p=0.022; 15% reduction per child). For internalising symptoms, playing alone more remained a significant moderator (p=0.014; 137% increase), all other previously significant moderators were no longer significant. Two additional moderators now showed significant pandemic effects: higher number of children living at home (p=0.009; reducing 25% per child) and having a BPL card (p=0.003; 20% increase).

## Discussion

Using an analysis that accounts for age-related changes in symptoms we provide the first prospective evidence of the impact of the pandemic on young preschool children’s mental health from a large birth cohort of Indian families. Overall, the pandemic was associated with a small and marginally significant increase in emotional problems. However, there was potentially important underlying variation in the behavioural and emotional impact on the child. Adjusting for age trends uniform across values of the moderator, family experience of greater COVID-19-related negative impacts, experiencing a higher number of COVID-19-related life events and the child spending more time playing alone were all associated with significant increases in child emotional and behavioural problems during the pandemic. Spending more time playing with a mobile phone, and maternal perceived stress are each associated with increases in emotional problems, whereas for behavioural problems, children whose mobile phone use did not increase during the pandemic showed an improvement in symptoms. Number of children in the household, parental job loss during the pandemic, previous partner violence, low socioeconomic status and child gender did not moderate the change in symptoms.

We tested for the possibility that age trends might differ according to the values of the moderator, but findings indicated that this was not common. Age by moderator interaction terms were statistically significant for only 3 of the 12 moderators; the number of other children living in the family home (in relation to behavioural and emotional problems), extent of TV watching and having a BPL card (in relation to emotional problems). The addition of these age by moderator interaction terms into each model typically changed little the *pattern* of moderation of pandemic effect estimates, although they did nonetheless have a marked impact on their apparent significance due to constraints on power. COVID-19-related negative impact remained a significant moderator of the pandemic effect on behavioural problems, and the child spending more time playing alone remained a significant moderator for emotional problems. Maternal perceived stress became a significant moderator for behavioural problems, rather than for emotional problems (as previously). These secondary analyses also revealed strengthened evidence for a protective effect of having other children living at home, for both behavioural and emotional problems. Finally, having a household income below the poverty line emerged as a significant moderator conferring risk for heightened emotional problems at follow-up.

There are few existing studies worldwide with preonset and postonset pandemic data able to assess the impact of the pandemic on emotional and behavioural problems in preschool children. In a sample of Spanish preschool children aged 4–6 years (n=157, 58.8% response), Alonso-Martínez *et al*^1^ reported an increase in behavioural and emotional symptoms from 3 to 6 months prior to the pandemic to immediately postonset during the lockdown. However, in another small sample (n=113, 67.7% response rate) in Spain, there was no change in behavioural problems from immediately preonset to 6 weeks postonset of the pandemic in children aged 3–6 years.^3^ Both studies used validated but not widely used measures of child symptoms. In US children of hourly service workers (n=561), Gassman-Pines^2^ reported that child uncooperative behaviour and worry assessed using daily reports did not significantly increase from immediately prior to during the pandemic.^2^ However, the number of COVID-19-related family hardships (eg, job loss, income cut) was strongly associated in cross-section with increased child problems during the pandemic. In a large population-based Brazilian birth cohort (n=2183, 50.1% response)^4^ assessed 6–14 months prior to the pandemic, at mean age 4 of years, and 6 months postpandemic, no overall increase in child emotional and behavioural problems on the Strengths and Difficulties Questionnaire—SDQ^25^ was found. However serious financial problems, food shortages, increased conflict in adult relationships, parenting problems and child worries about food availability during the pandemic were all associated with an increase in child problems. In a sample of 2340 (43.9% response rate) Chinese 3–4-year olds who were assessed in the 3 months prior to the pandemic onset and again 9 months postonset, Ding and colleagues^26^ examined change in symptoms for three groups of parent-rated COVID-19 impact (low impact, moderate impact and severe impact). Total problems on the SDQ and anxiety on the Spence Anxiety Scale^27^ significantly improved in the groups with low and moderate impact, and the trend was to improve in the severe impact group but this was non-significant. However, severe impact was associated in cross-section with increased symptoms postonset of the pandemic.

Collectively the evidence supports little to no overall impact of the pandemic on preschool children’s mental health symptoms. However, as the age range of the children in these samples varied from 3 to 6 years any change from pre-to-during the pandemic is confounded with normative developmental change in behavioural and emotional symptoms. Reports from multiple large datasets from high income countries have shown that the normative trajectory of behavioural problems is to decrease over the preschool period.^28^,^30^ The pattern of results for emotional problems is less clear, with most studies reporting an increase^28 29^ but others reporting a decrease^30^ over the preschool period. In the first prospective data over this age range in an Indian sample, we showed a very similar pattern of decreasing behavioural problems. Emotional problems also very slightly decreased. Importantly, by accounting for these normative developmental changes in our analysis, we provide robust evidence that in the overall sample there was minimal change associated with the pandemic. However, this small increase masked more substantial differences for groups linked to COVID-19-associated adversities.

In reviewing our findings next, we focus on results from our primary analyses where secondary analysis showed no significant age by moderation interaction was evident, so age trends did not differ across levels of the moderator variable. For the three moderators (number of children living in the family home, extent TV watching and having a BPL card) where significant age by moderator interactions were subsequently evident, we focus on those findings. Overall, and consistent with Murray *et al*,^4^ we found markedly increased child emotional and behavioural problems in families with a greater experience of COVID-19-related risks. Specifically, parental reports of more severe COVID-19 impact (eg, difficulties in accessing food and medicines, and adverse family conditions such as changes in noise levels) and the experience of higher numbers of COVID-19-related stressful events (eg, changes in childcare, separations from members of the family) were associated with an increase in child emotional and behavioural problems. Maternal perceived stress was associated with an increase in emotional problems. Pandemic-related impacts on the family may be particularly relevant for younger children who typically spend more time with and are more dependent on their parents. Especially in India, where relatives and extended families are commonly involved with shared childcare, the social isolation and travel restrictions led to hardships and changes in the broader family situation for many. Conversely, when COVID-19 impact on parental resources was lower, children may have benefitted from more time with parents and family during lockdown. In fact, we found behavioural problems, and, to a lesser extent, emotional problems decreased compared with the whole sample age-expected rates in families who experienced no COVID-19-related impacts and events.

An increase in screen time during lockdown periods has been documented by others,^31^ and we showed that increased play with a mobile phone was associated with an increase in child emotional problems. High levels of mobile phone use have been reported among children in India both in clinical and community samples^32^,^34^ and was a concern prepandemic.^35^ Mobile phones are commonly used for play and entertainment even with very young children including while feeding them. This may have increased during lockdown especially in low-income families where children had no access to other means of entertainment. The children in our study were also too young to have structured online schoolwork. The most common screen time use in India among children is with television as even low-income families have a television set, but in our study increased TV watching did not moderate mental health outcomes and in secondary analyses this remained non-significant.

Behavioural problems decreased compared with the whole sample age-expected rates in children who did *not* increase their mobile phone use. It is important to note that the study children were on average 4 years old (range 3–5) and an increase in mobile phone use as a focus for play at this age may be particularly concerning for social development. An increment in the time spent playing alone is another concern which may be particularly relevant for younger children who, in addition to being socially restricted due to COVID, could not engage with friends remotely. Social isolation and loneliness in children have both been linked to emotional problems^36^ and our finding that playing alone more was a significant moderator of emotional problems during the pandemic is consistent with this. This finding remained robust and significant after accounting for a significant age by moderator interaction. In line with this, our secondary analyses also revealed that having more children living in the family home was protective for both emotional and behavioural problems, which may well have ensured stimulation and mitigated against social isolation and boredom.

In adolescents, several studies from HIC have found girl’s mental health to be more negatively impacted by the pandemic^37 38^ although studies with mixed age ranges have found no gender difference in change^39^ which is consistent with the present findings. Evidence from HIC on whether families experiencing deprivation were more adversely affected is mixed.^38 40^ However, the large population-based Brazilian cohort study found low income and income loss were associated with a negative impact on child mental health.^4^ With fragile socioeconomic conditions in India, due to higher levels of relative poverty and many families relying on day wages for survival which were lost during lockdown, we expected impact to be greater in those experiencing deprivation but this hypothesis was not supported by our primary analysis where adjustment for age trends was made uniformly across different levels of the moderator. However, in our secondary analyses where a significant age by moderator interaction term was entered into the model, living below the poverty line did emerge as a significant moderator of the pandemic effect on emotional problems. Ownership of a BPL card identified a group experiencing deprivation who are also those given additional financial support by the government but even despite this the family may well have experienced more adversity.

Finally, our finding that the presence of previous family domestic violence did not moderate COVID-19 impact on child mental health was unexpected. Many have reported concerns in India about increased rates of partner violence during the pandemic,^41 42^ but ethical concerns about privacy and risk prevented us from enquiring about ongoing domestic violence in our telephone interviews with mothers during the pandemic. Future assessment waves with planned retrospective reporting of violence covering these periods may help to re-examine this question. Furthermore, in our analyses, we only examined the presence or absence of violence from birth to age 2, but the severity or frequency of violence may be most important for impact.

### Strengths and limitations

This paper highlights the methodological challenge of estimating variation in the impact of a universally experienced event like the pandemic in the context of typical preschool developmental change. One of the main strengths of this study is the longitudinal design with measurements prepandemic and during pandemic in a general population multi-year cohort of preschool-aged children in urban Bangalore, India. Our primary analysis took account of both the inevitable age-related changes in behavioural and emotional symptoms and the associations between COVID-19 moderators and prior functioning. This is important as the experience of adversities during the pandemic is related to a family’s general tendency to experience adverse events (eg, a family in a pre-existing difficult financial situation will be more likely to experience additional COVID-19 financial impacts). Nonetheless, our secondary analyses revealed that pandemic moderation estimates can also be confounded by developmental age trends that differ by the moderator-defined subgroups, increasing uncertainty. Despite these strengths of our analytic approach, we have not attempted to rule out possible reverse causation, from psychopathology to moderator, nor possible measurement method effects of having used the same reporter for outcomes and moderators.

A further strength is that the sample is largely representative of the population from which it was drawn, with similar maternal education level and religion to national data from 2015 to 2016 (National Family Health Survey—NFHS-4^24 43^). Slightly more of the sample possessed a BPL card than the general population report in 2017 (67% vs 50%^41^). Finally, compared with most COVID-19 follow-up surveys (~40–50%), we achieved a high response rate (78.0%).

A limitation may have arisen when estimating pandemic-associated change was by assuming typical maturational age-related change was linear within the log-link of the Poisson regression model. While alternative assumptions will yield different estimates for the overall change due to the pandemic, the identification of the important moderators is likely to be robust. We chose to plot the moderation effects of continuous variables at the extremes of the moderator range (rather than using mean±SD) to prevent the plots from becoming overcrowded with all the scatter points included. The assessment of family income and domestic violence in our study, as in all studies, could be prone to social desirability bias, with over and under-reporting, respectively. Although families were approached in random order in our COVID-19 follow-up phase, it is possible that families facing the COVID-19 adversities might have taken longer to contact, in which case child age at assessment could have been conflated with vulnerability associated with COVID-19 adversities. Although we found that child age and COVID-19 vulnerabilities were not correlated, this possibility cannot be ruled out.

Due to the need to keep the telephonic follow-up during the COVID-19 pandemic as brief as possible to minimise burden, we selected a subset of items from the CBCL externalising and internalising scales rather than using the full validated scales over the phone. These brief scales showed good psychometric properties and very high correlations with full scales at age 2 in India, but this may limit comparability to other studies which used the full scales. Finally, due to the severe stigma surrounding COVID-19 in India during the first wave of the pandemic, we were unable to record COVID-19 infection to collect data on family members acquiring the infection to examine whether this moderated impact.

## Conclusion

We report the first empirical study of COVID-19 impact on preschool mental health in India. Our findings are consistent with a growing literature from HIC and LMIC suggesting little to no overall impact of the pandemic on preschool-aged child mental health. However, our findings show that the impact of the pandemic was hugely variable and dependent on the experience of family adversities, with inequality in this respect. We found major differences in the impact on child mental health associated with COVID-19-associated adversities. Families who have been markedly affected will require continued support not just in managing the practical impacts but also in supporting their child’s emotional and behavioural problems over time. Children who were preschool age during the early phases of the pandemic will now be starting school and teachers should be supported in identifying ongoing emotional and behavioural problems. Without policy and practice initiatives designed to meet these additional mental health needs, such differences stand to exacerbate India’s mental health burden for years to come. Child mental health services in public health settings are limited and often handle only severe problems.^44^ However, the school mental health programme is more well developed especially in urban settings and may provide a platform for early detection of child emotional and behavioural problems and intervention.^45 46^ This approach is also likely to positively impact later educational outcomes for these children. Policy messages to reduce any ongoing negative impact of the pandemic should include advice on minimising mobile phone use by young children for play and the minimising the amount of time spent playing alone. From a research perspective, BCHADS is the only birth cohort with a focus on child mental health in India, so it is imperative to follow-up these children to establish the longer-term impacts of the pandemic throughout their development.

## supplementary material

10.1136/bmjph-2024-001209 online supplemental file 1

## Data Availability

No data are available.
