# Supplementary material for: Inequalities in COVID-19 impact on preschool mental health in India: key moderators of adverse outcome
Source: BMJ Public Health. 2024 Dec 12;2(2):e001209. doi: 10.1136/bmjph-2024-001209 (PMC11816102; doi:10.1136/bmjph-2024-001209)
Supplement: online supplemental file 1 [file bmjph-2-2-s001.docx]

# Inequalities in COVID-19 impact on pre-school mental health in India: key moderators of adverse outcome: Supplementary Appendices.

Helen Sharp, Department of Primary Care and Mental Health, Institute of Population Health, University of Liverpool, UK. hmsharp@liverpool.ac.uk

Nicky Wright, Department of Psychology, Manchester Metropolitan University, UK. Nicky.Wright@mmu.ac.uk

Laura Bozicevic, Department of Primary Care and Mental Health, Institute of Population Health, University of Liverpool, UK. lauraboz@liverpool.ac.uk

Thirumalai A. Supraja, Department of Psychiatry, Kasturba Medical College, Manipal Academy of Higher Education, Manipal, India. supraja.t.a@gmail.com

Andrew Pickles, Institute of Psychiatry, Psychology & Neuroscience, King’s College London, UK. andrew.pickles@kcl.ac.uk

Jonathan Hill, Department of Psychology, Manchester Metropolitan University, UK. jonathan.hill@mmu.ac.uk

Prabha S Chandra, National Institute of Mental Health and Neurosciences, Bangalore, India. chandra@nimhans.ac.in

**Correspondence**

Correspondence to Prof Helen Sharp, Department of Primary Care and Mental Health, University of Liverpool, Eleanor Rathbone Building, Bedford Street South, Liverpool, L69 7ZA, UK. Tel: +44 (0) 151 794 2000. Email: Hmsharp@liverpool.ac.uk

# APPENDIX 1: Participant Flow Chart

# Figure S1 Participant Flow Chart.


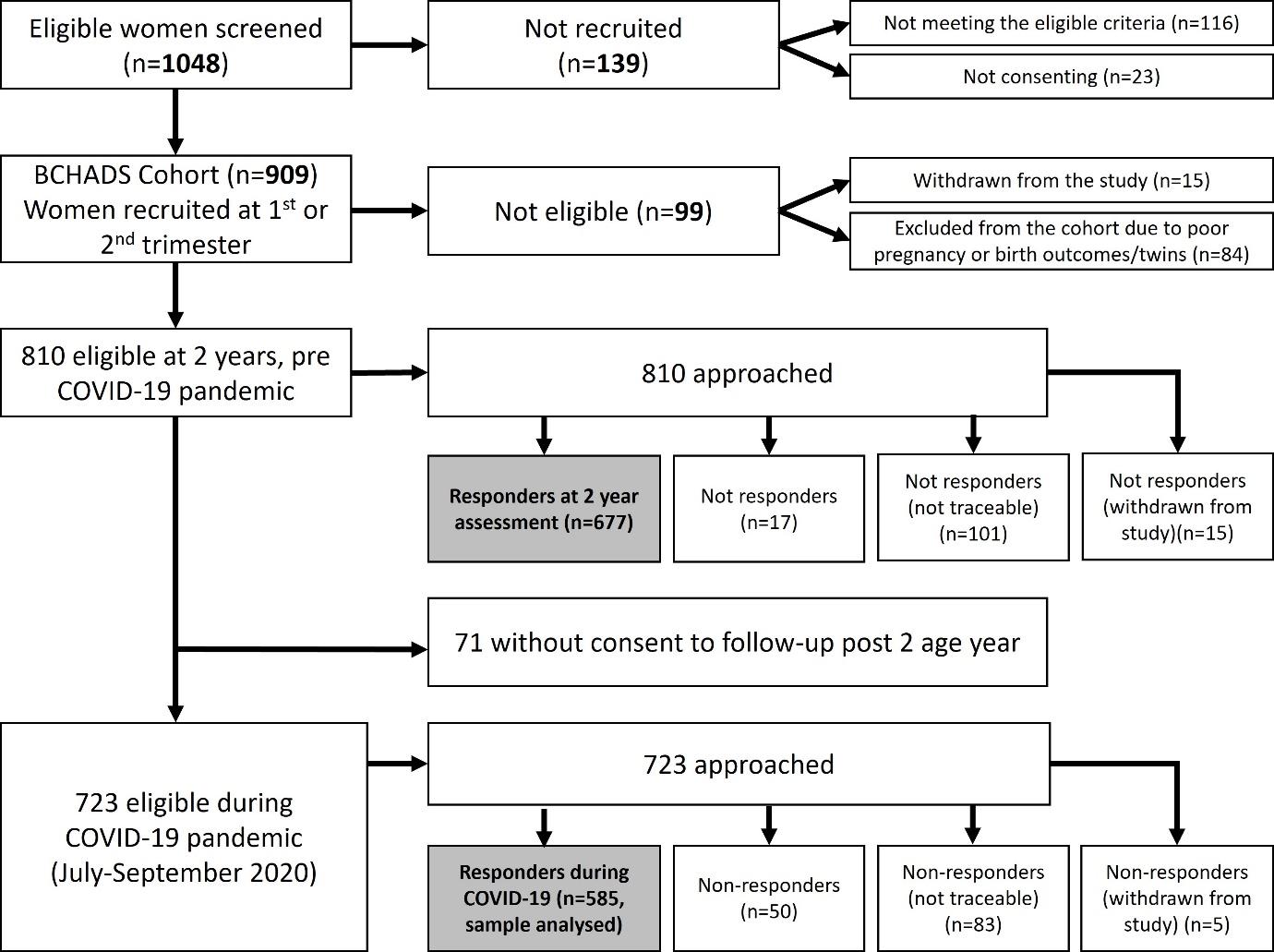


# APPENDIX 2: Description of the generation of the short CBCL scales used at T11

Items were selected on the basis of three separate factor analyses of the previously collected data from the pre-school CBCL in the BCHADS sample at age 2 (N=677), and data from a sister cohort in the UK (the Wirral Child Health and Development Study (WCHADS)) at age 2.5 years (N=253) and the WCHADS sample at age 5 (N=770).

From the original full Internalizing broadband scale, 12 items loaded on the ‘internalizing factor’ in all three samples above the a priori threshold set of .50. 6/12 of these were ‘Withdrawn (WD)’ items, 3/12 were ‘Anxious/Depressed (AD)’ items, 3/12 were ‘Emotion Regulation’ (ER) items. In order to ensure subdomains of internalizing problems were similarly represented in the short form for the current study, 2 WD items were then replaced with 2 AD items that loaded >.50 on at least two out of three factor analyses. 2 ER items were also added on this basis. The resultant 13 item short form was therefore comprised of 3 WD items, 5 AD items and 5 ER items. A similar approach was taken for the Externalizing broadband scale. The short-form comprised 12 items that loaded on an Externalizing Factor at >.50 at 2.5 y in BCHADS, 2.5 in WCHADS and age 5 in WCHADS plus 3 items which loaded at >.50 in two out of three Factor Analyses. The resultant 15 item short-form was therefore comprised of 13 items assessing ‘Aggressive Behaviour’ and 2 items representing ‘Attention problems’ which broadly reflects the weight given to these two domains within the full externalizing broadband scale.

Confirmatory factor analysis using Mplus version 8 (1) was next used to test the fit of the brief scales with internalizing and externalizing estimated as two correlated factors. The model showed good fit at age 2 (RMSEA=.038 [95% CI .033 - .042), CFI= .911, TLI= .903) and during the COVID follow-up assessment (RMSEA= .063, CFI=.914, TLI=.906). The brief scales showed good internal consistency at age 2 in BCHADS (alpha = .68 and .82, for internalizing and externalizing respectively) and the respective short form subscale total scores correlated with total internalizing (rho=.84, p < .0001) and externalizing scales (rho = .96, p < .0001). The internal consistency at follow-up for these short form subscales was also good at alpha = .80 and .83, for internalizing and externalizing respectively. On the basis of these analyses, and for the purposes of examining change in mental health difficulties over time in India, we therefore felt confident in using total scores from the short form of the CBCL externalizing and internalizing subscales at 2 years and follow-up during COVID.

**REFERENCE**

1. Muthén LK, Muthén B. Mplus User’s Guide (Eighth ed.). Los Angeles, CA: Muthén & Muthén; 1998-2017.

# APPENDIX 3

# Table S1

Covid-19 impact, perceived stress, and life events scales.

| **COVID-19 impact scale** | | | | | |
| --- | --- | --- | --- | --- | --- |
|  | **Not at all** | | **A little** | **Moderately** | **Severely** |
| 1. How much has/did the Covid-19 pandemic affected the availability of food for your family due to store closures (e.g., groceries, vegetables, fruits)? |  | |  |  |  |
| 2. How much has/did the Covid-19 pandemic affected the availability of food for your family due to lack of money (e.g., groceries, vegetables, fruits)? |  | |  |  |  |
| 3. During the Covid-19 pandemic, has/did the health of any family member been affected due to the lack of access to healthcare services? |  | |  |  |  |
| 4. How much has/did the Covid-19 pandemic affected your ability to buy medicines? |  | |  |  |  |
| 5. Due to the Covid-19 changes, have there been any difficulties in family relationships? |  | |  |  |  |
| 6. Due to the Covid-19 changes, has any other family member been feeling more stressed / tensed? |  | |  |  |  |
|  | **Less than usual** | | **The same** | **More than usual** |  |
| 7. During the COVID problem, has your home been more crowded than usual? |  | |  |  |  |
| 8. During the COVID problem, have the noise levels at home changed? |  | |  |  |  |
| **COVID-19 perceived stress scale** | | | | | |
|  | **Not at all** | | **A little** | **Moderately** | **Severely** |
| 1. Due to the Covid-19 changes, have you been feeling more stressed / tensed? |  | |  |  |  |
| 2. During the COVID problem, how worried have you been about yourself, your family catching COVID-19? |  | |  |  |  |
| 3. During the COVID problem, how worried are you about availability of food and other essential items? |  | |  |  |  |
| **Lockdown perception** |  | |  |  |  |
| Which of the following statements reflects your position best?  A. For you the effects of lockdown are ENTIRELY negative  B. For you the effects of lockdown are BOTH negative and positive  C. For you the effects of lockdown are MORE positive than negative  D. Your life has NOT really changed that much as a result of lockdown |  | |  |  |  |
| **Covid-19 life events scales**  *(Since the COVID-19 emergency started, have you and your family experienced any of the following things?)* | | | | | |
|  | **Yes** | | | **No** | |
| 1. The STUDY child not being able to see one of their parents that they would normally have regular contact with |  | |  |  |  |
| 2. Needing to take supplies to a relative/friend who is self-isolating (NOTE: Self-isolating means staying at home and avoiding contact with people outside their household) |  | |  |  |  |
| 3. Having to sort out different childcare for your children (e.g. hiring a caretaker, sharing this responsibility with your spouse, leaving the child with a relative) |  | |  |  |  |
| 4. Having to cancel a holiday |  | |  |  |  |
| 5. Having to cancel another important social event |  | |  |  |  |
| 6. Having to cancel a house sale/buy |  | |  |  |  |
| 7. Being separated from your husband |  | |  |  |  |
| 8. Being separated from one or more of your children |  | |  |  |  |
| 9. Being separated from a friend or family member you are very close to |  | |  |  |  |
| 10. Having to stop treatment for a physical problem (e.g. physiotherapy) or not being able to have treatment you have been waiting for (e.g. minor surgery) |  | |  |  |  |
| 11. Having to stop treatment for a psychological problem (e.g. therapy, AA groups) |  | |  |  |  |
| 12. You are pregnant and your antenatal care is affected |  | |  |  |  |
| 13. Not being able to care for elderly relatives in the usual way |  | |  |  |  |
| 14. Looking after a person with special needs, disabilities or a vulnerable adult at home without usual support |  | |  |  |  |
| 15. Difficulty getting medicines |  | |  |  |  |
| 16. Difficulty getting essential supplies (e.g. food and cleaning products) |  | |  |  |  |
| 17. Intolerable or difficult overcrowded living situation |  | |  |  |  |
| 18. Being in the same house on lockdown with a family member you do not get on well with |  | |  |  |  |
| 19. Struggle keeping your children occupied |  | |  |  |  |
| 20. Not being able to get through to see a doctor when you need to |  | |  |  |  |
| **Changes due to COVID-19** |  | |  |  |  |
|  | **Yes** | | | **No** | |
| a. Have you lost your job? |  | |  |  |  |
| b. Have your husband lost his job? |  | |  |  |  |
|  | **Less than usual** | | **The same** | **More than usual** |  |
| *During Covid-19 how often has your child been doing the following activities compared to usual (before COVID-19 lockdown)?* | |  |  |  |  |
| a. Playing alone |  | |  |  |  |
| b. Watching TV |  | |  |  |  |
| c. Playing with the mobile phone |  | |  |  |  |

**Figure S2**

Association between perceived maternal stress and internalizing symptoms.


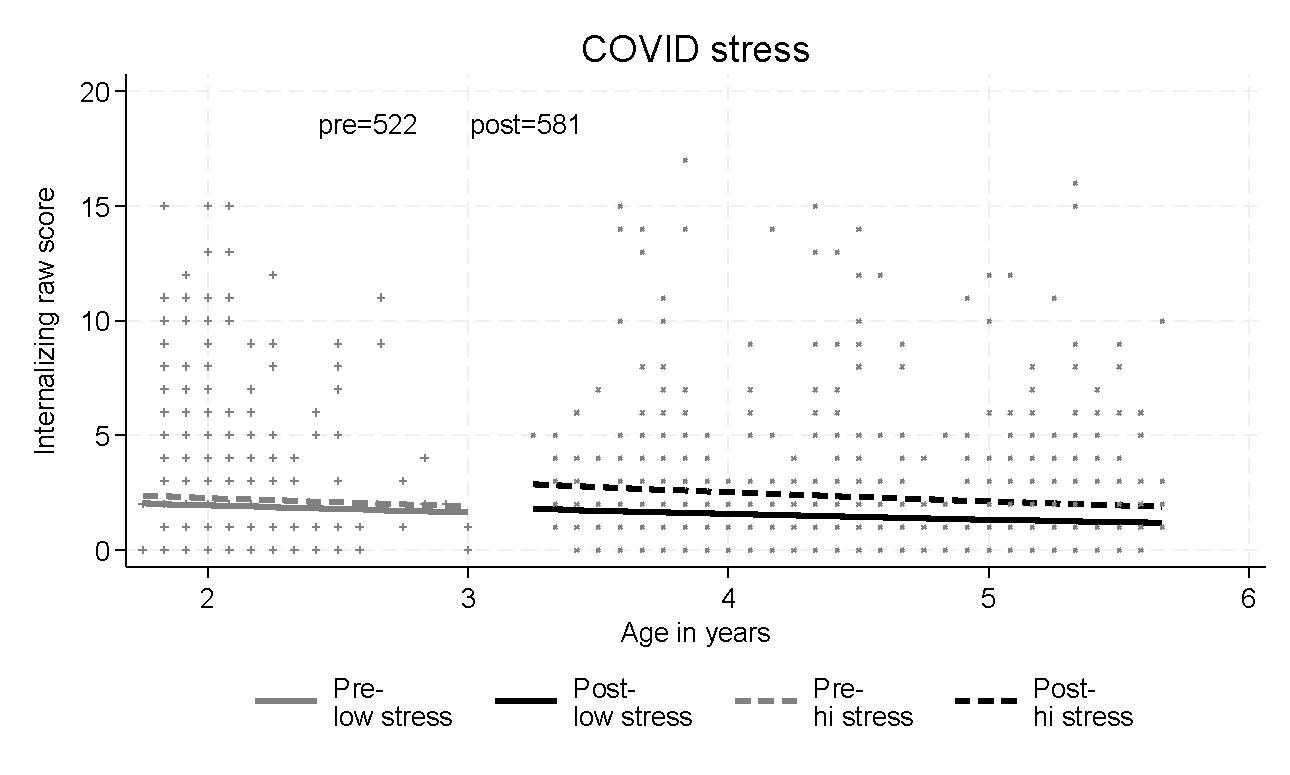


**APPENDIX 4:** Statistical model

# Primary Analyses: The fitted model is a form of repeated measures or seemingly unrelated regression model for two over-dispersed Poisson count-like variables, denoted y1 and y2. The correlation between measures is accounted for by the inclusion of a shared random effect or latent variable F. With a common age-trend over the two ages at each assessment, and ages centred around age 3 that lay between pre and post-pandemic assessments, pandemic effects are estimated as the difference in intercepts.

# E[y1] = exp(cons1 + b1.age1+ F), E[y2] = exp(cons2 + b1.age2+ F),

# F ~N(0,sd)

Pandemic effect = exp(cons2-cons1)

With moderator z, the model is extended with coefficient a1 estimating the difference pre-pandemic and b2 the additional post pandemic effect.

# E[y1] = exp(cons1 + b1.age1 + F), E[y2] = exp(cons2 + b1.age2 + F + b2.z)

F ~N( a1.z, sd)

Pandemic effect = exp(cons2 - cons1 + b2.z)

Secondary analyses: The model was further extended to allow for possible age trends that varied with z though the addition of an age by z interaction

# E[y1] = exp(cons1 + b1.age1+ F + b3.age1.z),

# E[y2] = exp(cons2 + b1.age2 + F + b2.z + b3.age2.z)

F ~N( a1.z, sd)

Pandemic effect = exp(cons2 - cons1 + b2.z)

**APPENDIX 5:** Results from extended model including sub-group moderated age-trends

Figures S3 to S6 present results analogous to those of the main paper

**Figure S3**

Model estimates of the decomposition of the change in child symptoms due to maturation and onset of the pandemic, overall and in COVID-19 impact groups.


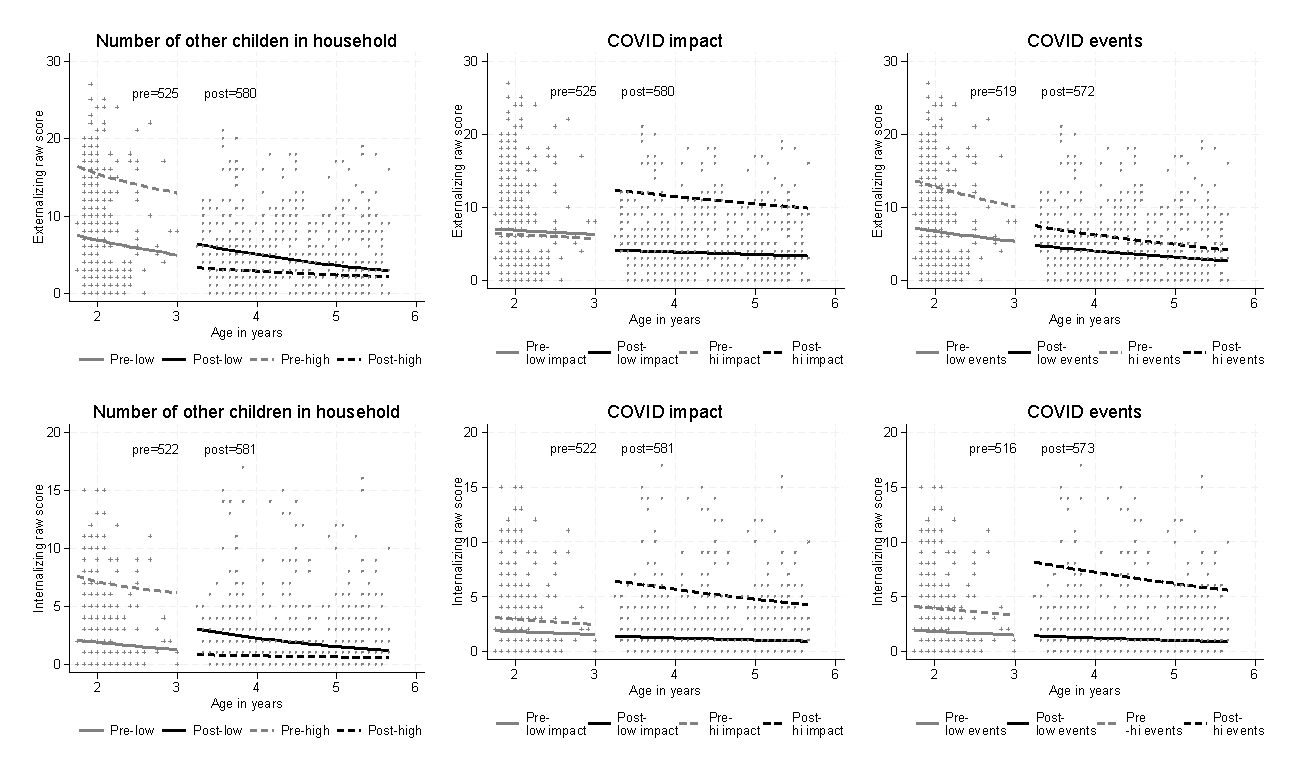


The Figure shows the model estimated systematic decline in symptoms with age within each moderator defined sub-group of the cohort pre- and during the pandemic, with a disjunction between the two reflecting the estimated impact of the pandemic.

**Figure S4**

Model estimates of the decomposition of the change in child symptoms due to maturation and to the onset of the pandemic, in high and low mobile phone use, playing alone and negative perception of lockdown groups.


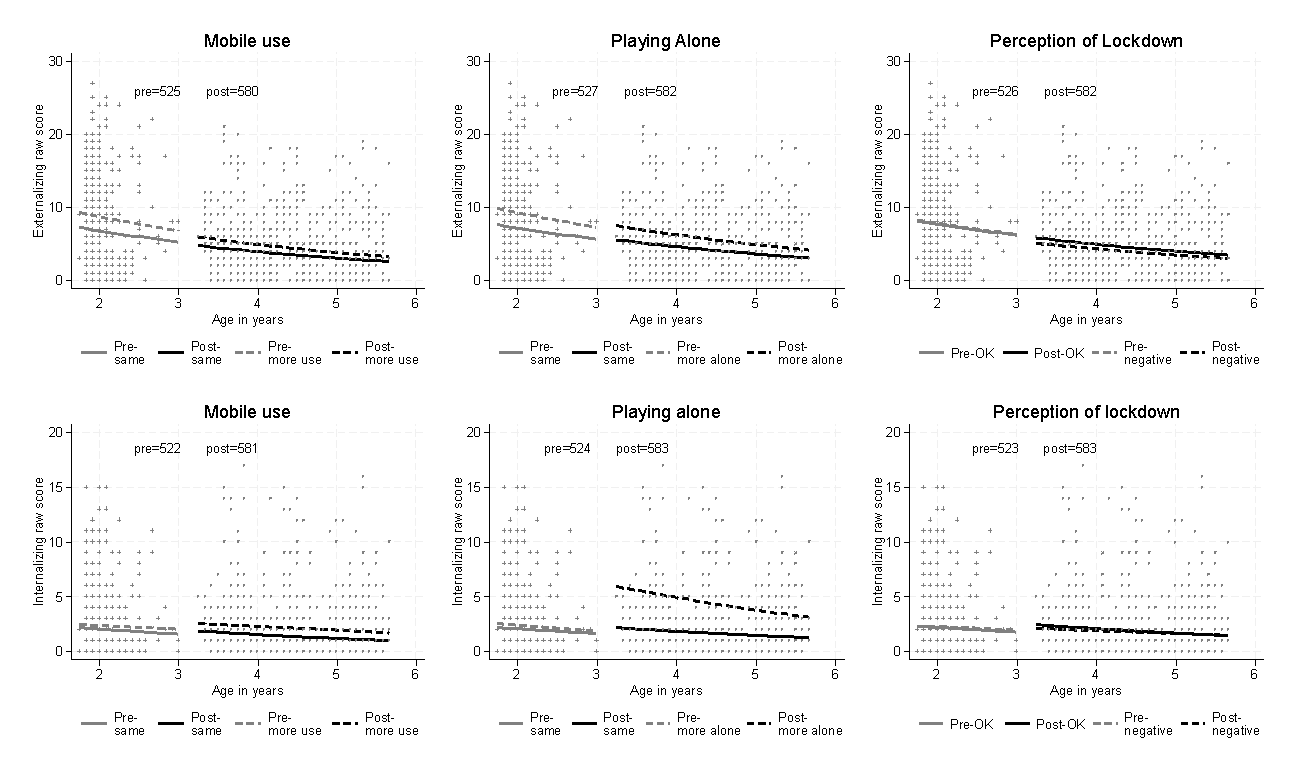


**Figure S5**

Child CBCL parent-rated externalizing score: estimates of change attributed to the pandemic from the extended model. For moderators treated as continuous group size denotes overall sample size. Moderator p-value denotes significance of moderator on pandemic effect and not significance of the difference from no-pandemic effect (null value=1) for the sub-group.


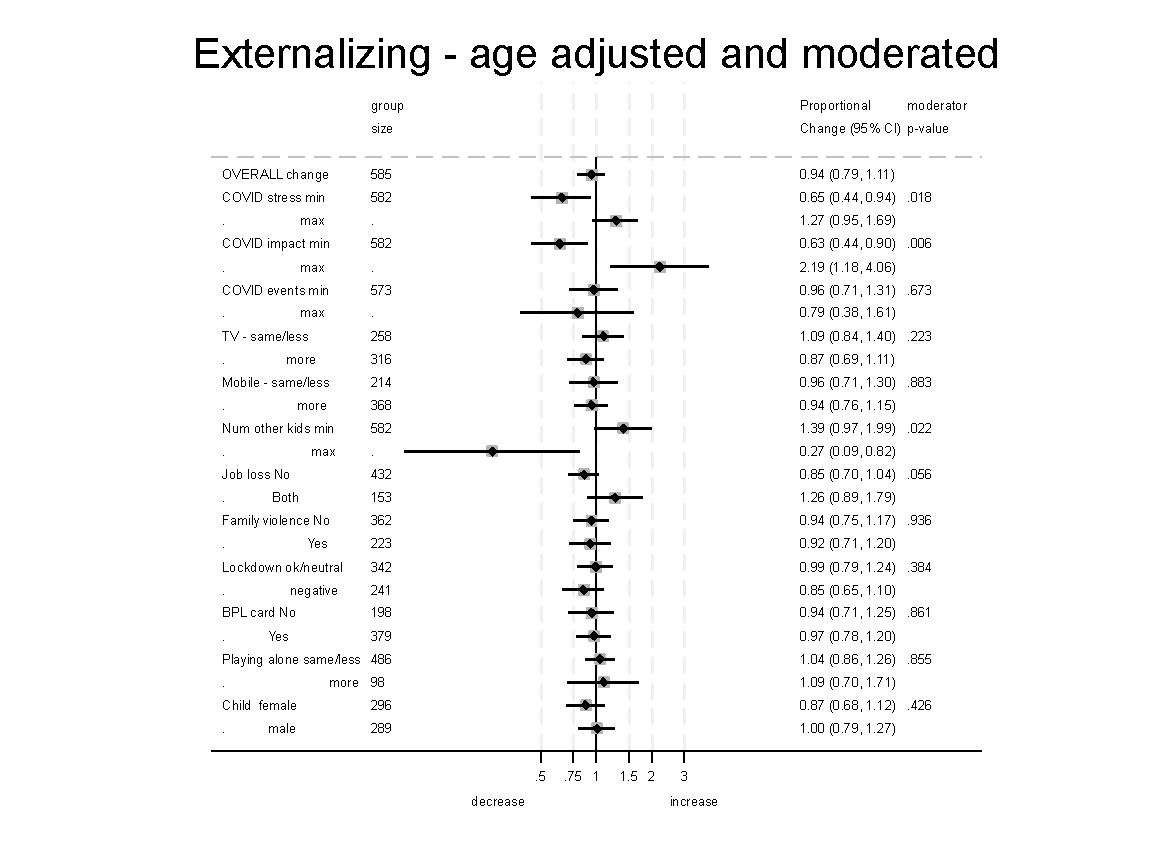


**Figure S6**

Child CBCL parent-rated internalizing score: estimates of change attributed to the pandemic from the extended model. For moderators treated as continuous group size denotes overall sample size. Moderator p-value denotes significance of moderator on pandemic effect and not significance of the difference from no-pandemic effect (null value=1) for the sub-group.


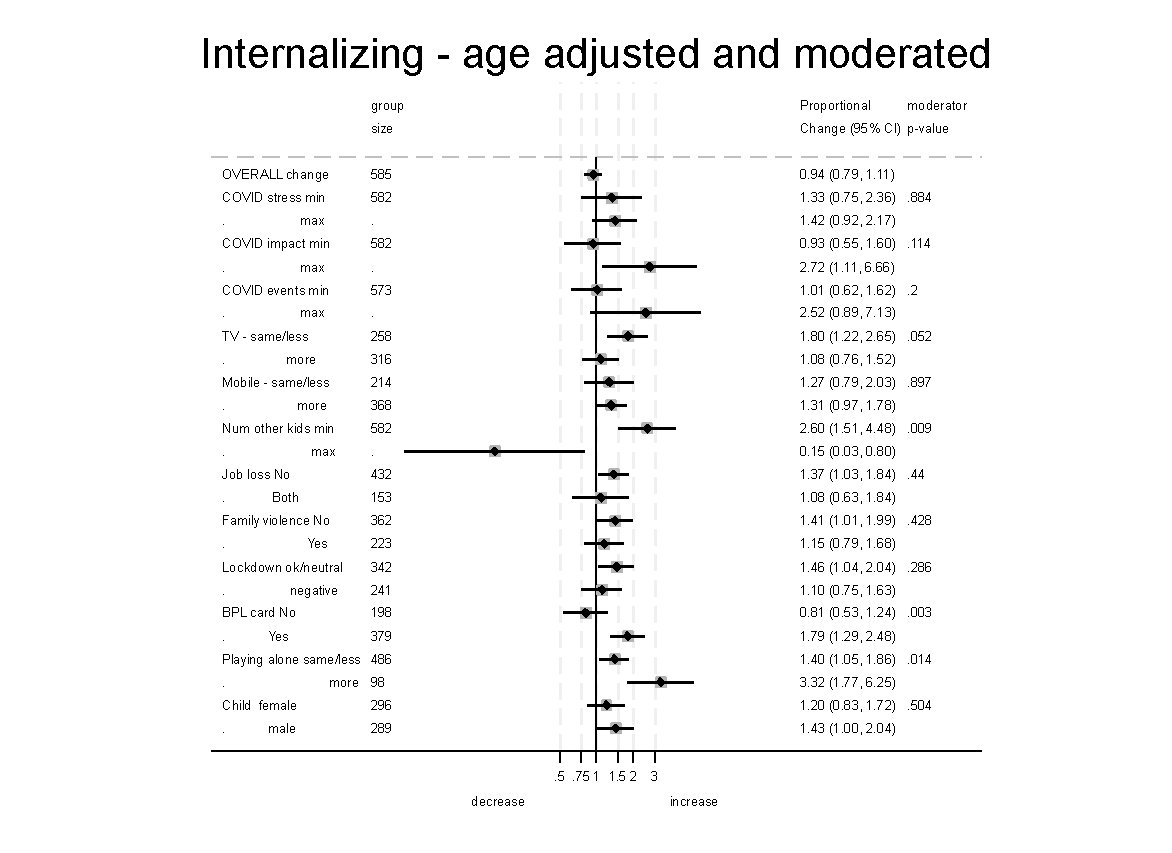


**APPENDIX 6:** Descriptive table in relation to those included and excluded from the analysis sample

**Table S2**

Demographic information of women excluded and included in the analyses

| **Variable** | **Excluded in Analysis Sample**  N (with var) Mean(SD) or % | | **Included in Analysis Sample**  N (with var) Mean(SD) or % | |
| --- | --- | --- | --- | --- |
| Education (yrs) | 322 | 3.07 (1.02) | 584 | 3.25 (1.00) |
| Marriage (yrs) | 324 | 3.09 (3.18) | 581 | 3.15 (3.11) |
| Age married | 323 | 22.87 (3.55) | 585 | 22.80 (3.96) |
| Gest-age (wks) | 180 | 38.48 (2.99) | 580 | 38.83 (2.47) |
| Log-income | 257 | 9.40 (0.59) | 527 | 9.37 (0.55) |
| Family type  Nuclear  Joint  Extended | 320 | 49%  42%  9% | 572 | 41%  50%  9% |
| Child sex  Male  Female | 165 | 52%  49% | 585 | 48%  51% |
| Religion  Hindu  Muslim  Christian | 322 | 82%  15%  2% | 581 | 84%  15%  0% |
| Marriage Type  Consanguineous  Non-consang. | 323 | 28%  72% | 581 | 27%  73% |

**Note**: Total number variations are due to the missingness of some of the demographic information for some of the participants
